# Extended letrozole regimen versus clomiphene citrate for superovulation in patients with unexplained infertility undergoing intrauterine insemination: A randomized controlled trial

**DOI:** 10.1186/1477-7827-9-84

**Published:** 2011-06-21

**Authors:** Usama M Fouda, Ahmed M Sayed

**Affiliations:** 1 Department of Obstetrics and Gynecology, Faculty of Medicine, Cairo University, Cairo, Egypt

## Abstract

**Background:**

The aim of this randomized controlled trial was to compare the efficacy of extended letrozole regimen with clomiphene citrate in women with unexplained infertility undergoing superovulation and intrauterine insemination (IUI).

**Methods:**

Two hundred and fourteen patients with unexplained infertility were randomized into two equal groups using computer generated list and were treated by either letrozole 2.5 mg/day from cycle day 1 to 9 (extended letrozole group, 211 cycles) or clomiphene citrate 100 mg/day from cycle day 3 to 7 (clomiphene citrate group,210 cycles). Intrauterine insemination was performed 36 to 40 hours after HCG administration.

**Results:**

Both groups were comparable with regard to number of mature follicles (2.24 +/- 0.80 Vs 2.13 +/- 0.76) and the day of HCG administration. Serum estradiol was significantly greater in clomiphene citrate group (356 +/- 151 Vs 822 +/- 302 pg/ml, P = < 0.001) and the endometrial thickness was significantly greater in extended letrozole group (9.10 +/- 1.84 Vs 8.18 +/- 1.93 mm, P = < 0.001).The pregnancy rate per cycle and cumulative pregnancy rate were significantly greater in extended letrozole group (18.96% Vs 11.43% and 37.73% Vs 22.86%, respectively).

**Conclusion:**

The extended letrozole regimen had a superior efficacy as compared with clomiphene citrate in patients of unexplained infertility undergoing superovulation and IUI.

**Trial registration:**

ClinicalTrials.gov, NCT01232075

## Background

Unexplained infertility is one of the most frequent infertility diagnoses encountered by the gynaecologists. Various studies reported that 10 to 30% of infertile couples have unexplained infertility [1,2].

Superovulation and intrauterine insemination (IUI) is an effective treatment for women with unexplained infertility [3]. Superovulation increases the probability of pregnancy by increasing the number of oocytes suitable for fertilization or by correcting any subtle defect in ovulation. Furthermore, IUI increases the concentration of active motile sperms reaching the fallopian tubes and overcomes male factors or cervical factors of infertility not detected by conventional infertility tests [4].

For more than four decades, clomiphene citrate has been the first line therapy for induction of ovulation in women with anovulatory infertility and for superovulation in couples with unexplained infertility, mild endometriosis and mild male factor of infertility. Clomiphene citrate is cheap, orally administered and is associated with very low risk of high-order multiple gestation and severe ovarian hyperstimulation syndrome (OHSS)[5,6]. However, clomiphene citrate induces prolonged estrogen receptors depletion and therefore exerts antiestrogenic effect on estrogen target tissues as endocervix and endometrium. Several studies revealed that clomiphene citrate has a deleterious effect on cervical mucus quantity and quality and endometrial development resulting in decreased uterine blood flow, endometrial thinning, luteal phase defect and implantation failure [7,8].

During the past decade, letrozole (aromatase inhibitor approved by FDA for the treatment of postmenopausal women with breast cancer) has been successfully used for induction of ovulation in anovulatory patients with polycystic ovary syndrome (PCOS) and for augmentation of ovulation in ovulatory women [6,9]. In contrast to clomiphene citrate, letrozole is rapidly eliminated from the body and does not deplete estrogen receptors and therefore has no adverse effect on endometrium or endocervix [10,11].

Several studies revealed that letrozole can be used as an alternative to clomiphene citrate for superovulation in patients with unexplained infertility [12,13]. A meta-analysis of seven randomized controlled trials comparing aromatase inhibitors (letrozole or anastrozole) with clomiphene citrate for superovulation in patients with unexplained infertility undergoing IUI revealed that the pregnancy rate was comparable between both management options [14].

The optimal dose and duration of letrozole administration for superovulation in patients with unexplained infertility are still not clear. In various studies reporting the use of letrozole for superovulation, letrozole was administered from cycle 3 to 7 with daily dose ranging from 2.5 mg to 7.5 mg [6]. In a randomized controlled trial, Al-Fadhli et al found that the pregnancy rate was significantly higher in patients with unexplained infertility treated with 5 mg/day compared with those treated with 2.5 mg/day [15]. On the other hand, a recent randomized controlled trial revealed that the pregnancy rates were comparable in three groups of patients with unexplained infertility treated with three different doses of letrozole (2.5, 5 or 7.5 mg/day) [16].

In a recent study, Badawy et al reported that the extended letrozole regimen (2.5 mg/day from cycle day 1 to10) resulted in higher pregnancy rate compared with short high dose letrozole regimen (5 mg/day for 5 days) in clomiphene-resistant women with polycystic ovary syndrome [17].

The aim of this randomized controlled trial was to compare the efficacy of extended letrozole regimen (2.5 mg/day from cycle day 1 to 9) with clomiphene citrate (100 mg/day from cycle day 3 to 7) in women with unexplained infertility undergoing superovulation and IUI.

## Methods

This prospective, assessor blinded, allocation concealed, multicenter, two arm randomized controlled trial included 214 women (421 cycles) with unexplained infertility among those attending the outpatient clinic of Cairo university hospital and Ahmed Elgazzar hospital, Cairo, Egypt between September 2008 and December 2010. The study protocol was approved by ethics committees of both hospitals. The patients were counselled about the benefits and risks of letrozole and clomiphene citrate therapy and informed consent was obtained before randomization.

Patients with unexplained infertility and at least one year of infertility were included in the study. All the patients had patent fallopian tubes detected by hysterosalpingography and/or laparoscopy, normal ovulation confirmed by midluteal progesterone level more than 5 ng/ml and normal hormonal profile (FSH, LH, prolactin and TSH) in the early follicular phase. All the male partners had normal semen analysis according to WHO criteria [18].

Our exclusion criteria were patients with irregular cycles, ovarian cysts, PCOS, endometriosis, FSH >10 mIU/ml, age less than 18 years or more than 37 years, previous IUI cycles and liver or kidney diseases.

Patients were randomly allocated to extended letrozole group or clomiphene citrate group using a computer generated randomization list and sequentially numbered opaque sealed envelopes, each containing the allocation information written on a card. Envelopes were opened sequentially by a study nurse to allocate patients to the assigned group. The extended letrozole group included 107 patients (211 cycles) who were treated with letrozole (Femara; Novartis pharma AG, Basle, Switzerland) 2.5 mg/day from cycle day 1 to 9. The clomiphene citrate group included 107 patients (210 cycles) who were treated with clomiphene citrate (Clomid; Aventis pharma S.AE, Global Napi pharmaceuticals, Cairo, Egypt) 100 mg/day from cycle day 3 to 7. All the patients underwent 1 to 3 IUI cycles.

Human chorionic gonadotropin (Pregnyl; N.V. Organon, Oss, Holland) (10.000 IU/I.M) was administered to trigger ovulation when at least one follicle measured more than 18 mm in mean diameter. Intrauterine insemination was performed 36 - 40 hours after HCG injection using soft tip catheter. Serum B-subunit HCG was measured 2 weeks after IUI to diagnose pregnancy. Ultrasound examination was performed 5 weeks after IUI to confirm the presence of fetal cardiac activity and to exclude ectopic pregnancy.

Starting from cycle day 9, ultrasound scans were repeated daily to monitor follicle growth. Serum estradiol and endometrial thickness were measured on the day of HCG administration. Endometrial thickness was measured at the greatest diameter perpendicular to the midsagittal plane in the fundal region.

The doctor responsible for ultrasound examination (U.M. Fouda) was blinded to the treatment protocol. The patients and the other caregivers at both hospitals were not blinded to the treatment protocol.

The primary end point was the clinical pregnancy rate (presence of gestational sac in uterine cavity detected by transvaginal ultrasound). The secondary end points were the number of follicles with mean diameter more than 18 mm, serum estradiol and endometrial thickness on the day of HCG administration, ongoing pregnancy rate (pregnancies continued beyond 20 weeks gestation), miscarriage rate(termination of pregnancy before the 20^th ^gestational weeks), ectopic pregnancy rate and multiple pregnancy rate.

### Sample size calculation

The most recent and largest randomized controlled trial at the time of study design was used for sample size calculation. Badawy et al reported that the cumulative clinical pregnancy rate in 207 patients (404 cycles, 1.95 cycle/woman) with unexplained infertility undergoing superovulation and IUI was 35.6% when clomiphene citrate was used for superovulation [19]. We considered that 20% increase in cumulative clinical pregnancy in favour of extended letrozole regimen would be of clinical significance. To detect 20% difference in cumulative pregnancy rate between extended letrozole group and clomiphene citrate group (55.6% Vs 35.6%), each group should include 97 patients to give the study 80% power at the 5% significance level. We expected that the dropout incidence would be 10%, therefore 107 patients were included in each group.

### Statistical analysis

SPSS (Statistical Package for the Social Science; SPSS Inc., Chicago, IL, USA) statistical program for Microsoft Windows was used for statistical calculations. Comparison of quantitative variables between the study groups was done using Student's t-test for independent samples when normally distributed. For comparing categorical data, the Chi-square (χ^2^) test was performed. Yates correction equation was used instead, when the expected frequency was less than 5. A probability value (p value) less than 0.05 was considered statistically significant.

## Results

**Figure 1:**
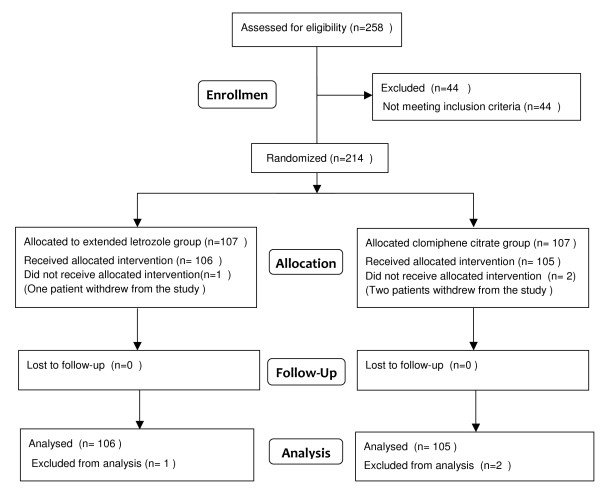
**Flow diagram of the study**.

A total of 214 patients (421 cycles) were recruited to the study with 107 patients randomized to each group. Two patients in clomiphene citrate group and one patient in extended letrozole group withdrew from the study. The mean number of cycles per woman was 1.99 in extended letrozole group and 2 in clomiphene citrate group. The flow of patients through the study is shown in Figure 1.

Table 1 shows the demographic criteria of both groups. There were no significant differences between both groups with respect to age, body mass index (BMI), duration of infertility, percentage of patients with primary or secondary infertility and baseline hormonal profile.

**Table 1:** Patients characteristics

	Extended letrozole group (n = 107)	Clomiphene citrate group (n = 107)	P value
**Age (years)**	26.68 ± 3.51	26.13 ± 3.22	0.233
**Body mass index (Kg/m**^**2**^**)**	26.08 ± 3.55	25.24 ± 4.01	0.110
**Duration of infertility(years)**	3.69 ± 1.88	3.40 ± 1.62	0.229
**Primary infertility**	70/107(65.42%)	77/107(71.96%)	0.302
**Secondary infertility**	37/107(34.58%)	30/107(28.04%)	0.302
**Day 2 FSH(IU/L)**	5.71 ± 1.95	5.51 ± 1.85	0.451
**Day 2 LH(IU/L)**	4.94 ± 1.89	5.14 ± 1.94	0.443
**Day 2 Estradiol(pg/ml)**	53.21 ± 13.45	50.47 ± 12.08	0.119

Values are expressed as mean ± SD or n/n (%)

Both groups were comparable with regard to number of follicles > 18 mm on the day of HCG administration (2.24 ± 0.80 Vs. 2.13 ± 0.76, *P *= 0.154). Serum estradiol was significantly lower in extended letrozole group (356 ± 151 Vs. 822 ± 302 pg/ml, *P *= < 0.001) and the endometrial thickness was significantly greater in extended letrozole group (9.10 ± 1.84 Vs. 8.18 ± 1.93 mm, *P *= < 0.001) (Table 2).

**Table 2:** Intrauterine insemination cycle characteristics

	Extended letrozole group (n = 106)	Clomiphene citrate group (n = 105)	P value
**No. of cycles completed**	211	210	
**No. of follicles > 18 mm**	2.24 ± 0.80	2.13 ± 0.76	0.154
**Days of HCG administration**	12.35 ± 1.05	12.50 ± 1.10	0.132
**Endometrial thickness on HCG day (mm)**	9.10 ± 1.84	8.18 ± 1.93	<0.001
**Serum E**_**2 **_**on HCG day (pg/ml)**	356 ± 151	822 ± 302	<0.001

Values are expressed as mean ± SD

The pregnancy rate per cycle and the cumulative pregnancy rate were significantly higher in extended letrozole group compared with clomiphene citrate group (18.96% Vs 11.43% and 37.73% Vs 22.86%, respectively). Five spontaneous abortions occurred in extended letrozole group and 4 spontaneous abortions occurred in clomiphene citrate group. There were 4 twin pregnancies in extended letrozole group and 3 twin pregnancies in clomiphene citrate group. No cases with ectopic pregnancies or ovarian hyperstimulation syndrome were reported in both groups. All the neonates were examined by a paediatrician after delivery. No congenital anomalies were detected (Table 3).

**Table 3:** Pregnancy outcomes

	Extended letrozole group (n = 106)	Clomiphene citrate group (n = 105)	Odd ratio (95% CI)	P value
**No of completed cycles**	211	210		
**Clinical pregnancy/cycle**	40/211(18.96%)	24/210(11.43%)	1.81(1.05, 3.13)	0.031
**Cumulative pregnancy rate**	40/106(37.73%)	24/105(22.86%)	2.05(1.12, 3.73)	0.019
**Ongoing pregnancy rate**	35/106(33.02%)	20/105(19.05%)	2.09(1.11, 3.95)	0.02
**Abortion rate**	5/40(12.5%)	4/24(16.67%)	0.71(0.17, 2.97)	0.926
**Multiple pregnancy rate**	4/40(10%)	3/24(12.5%)	0.78(0.16, 3.82)	0.756
**Ectopic pregnancy rate**	0/40(0%)	0/24(0%)	NA	NA
**OHSS**	0/106 (0%)	0/105(0%)	NA	NA

Values are expressed as n/n (%)

CI = confidence interval, OHSS = ovarian hyperstimulation syndrome.

## Discussion

To the best of our knowledge, this is the first study comparing extended letrozole regimen with clomiphene citrate for superovulation in patients with unexplained infertility undergoing IUI. The results of our study revealed that the extended letrozole regimen has a superior efficacy as compared with clomiphene citrate.

Letrozole is a third generation, potent, reversible, non-steroidal aromatase inhibitor. Letrozole administration in early follicular phase blocks estrogen syntheses by inhibiting aromatase enzyme which catalyses the conversion of androstenedione and testosterone to estrone and estradiol. The drop in the circulating estrogens levels (produced by the ovary and by conversion of androgens in adipose tissues) and locally produced estrogens in brain releases the hypothalamo-pituitary axis from estrogenic negative feedback on FSH and LH release. The increase in FSH secretion stimulates the recruitment and growth of antral follicles [20]. Furthermore, letrozole causes temporary accumulation of androgens in the ovarian follicles by blocking the conversion of androgens to estrogens. The accumulated androgens may increase the sensitivity of the growing follicles to FSH by increasing the expression of FSH receptors [21].

Because letrozole has short half life (average 45 hours), its effect decreases during late follicular phase and therefore estradiol produced by growing follicles increases. The elevated estradiol levels suppress the release of FSH. The drop in FSH levels causes atresia of all follicles smaller than dominant follicle leading to mono-ovulation in most cycles. On the other hand, clomiphene citrate induces prolonged estrogen receptors depletion in the brain and therefore the increased estradiol produced by the growing follicles is not capable of central suppression of FSH release. This maintains the release of high levels of FSH throughout the follicular phase and therefore induces development of multiple follicles [22].

Although mono-ovulation is the main advantage of induction of ovulation with letrozole in patients with PCOS who are often hyperresponders and at high risk for OHSS, multiple ovulation is desired in patients with unexplained infertility undergoing IUI [9]. Several studies revealed that the number of mature follicles is an important predictor factor for the success of IUI cycle. Sikander et al reported that the pregnancy rate per cycle after IUI was 6.2%, 12.9% and 30% with one, two and three mature follicles developed respectively [23].

The majority of studies comparing clomiphene citrate with letrozole for superovulation in patients with unexplained infertility undergoing IUI revealed that although letrozole induced fewer mature follicles compared with clomiphene citrate the pregnancy rate was comparable between both management options[9,12]. We think that letrozole resulted in comparable pregnancy rate as clomiphene citrate, in spite of less number of mature oocytes induced, because it has no adverse effect on endometrium. Boa et al found that the markers of endometrial receptivity (HOXA10 and integrin alpha (v) beta (3)) in rats were suppressed by clomiphene citrate and not affected by letrozole [24]. Moreover, Cortinez reported that letrozole administration in infertile ovulatory women was associated with in-phase histological dating of endometrium and normal pinopode expression [10].

In the present study, the number of mature oocytes was similar between both groups and the pregnancy rate per cycle and cumulative pregnancy rate were significantly greater in the extended letrozole group (18.96% Vs 11.43% and 37.73% Vs 22.86%, respectively).

There is only one study which reported the use of extended letrozole regimen in induction of ovulation. In that study, 218 patients with clomiphene citrate resistant PCOS were randomized to receive letrozole 2.5 mg from cycle day 1 to10 or letrozole 5 mg/day from cycle day 1 to 5. Extended letrozole regimen resulted in more mature follicles and pregnancies than short letrozole regimen [17].

In natural cycles the rise of FSH levels during the luteal-follicular transition phase stimulates the recruitment and growth of a cohort of antral follicles. The increase in the estradiol produced by the growing follicles (mainly the dominant follicle) suppresses FSH levels below the threshold required for the growth of all follicles smaller than dominant follicle [25]. Meanwhile, the dominant follicle continues to grow until the ovulatory stage because it is more sensitive to FSH [26]. Badawy et al suggested that the extended letrozole regimen can maintain FSH levels above the threshold required for the growth of follicles smaller than dominant follicle (i.e. widen FSH window) and therefore induces multiple ovulation [17].

The endometrial thickness was significantly greater in the extended letrozole group. The results of our study are in agree with the results of Metwally and Casper and Sh Tehrani-Nejad et al [20,27]. On the other hand, other studies revealed that the endometrial thickness was comparable in patients treated with letrozole or clomiphene citrate [19,28]. In only one study, the endometrial thickness was significantly greater in the group of patients treated with clomiphene citrate [29].

In patients with unexplained infertility undergoing IUI, we think that the extended letrozole regimen is more effective than conventional short letrozole regimen because it induces multiple ovulation [17] and more effective than clomiphene citrate because it has no adverse effect on endometrium [20]. Several studies revealed that the combination of exogenous gonadotropins with letrozole increased the number of mature oocytes and therefore improved the outcome of IUI cycles in patients with unexplained infertility [6,12]. However, the addition of exogenous gonadotropins to letrozole increases the cost of IUI cycle and is associated with increased risk of higher order multiple pregnancy and ovarian hyperstimulation syndrome. Our study highlights the need for larger randomized controlled trials to determine whether the extended letrozole regimen should be the treatment of choice for patients with unexplained infertility undergoing IUI.

The results of the studies evaluating the safety of letrozole are contradictory. In 2005, Bilijan et al compared 150 babies born after letrozole therapy with 36000 babies born to low risk pregnant women. Although the general incidence of anomalies was not increased, the incidence of cardiac and bone anomalies was higher in letrozole group [30]. Subsequent study comparing 514 babies born after letrozole therapy with 36000 babies born after clomiphene citrate therapy revealed that letrozole therapy was not associated with increased risk of congenital anomalies [31]. In another study, Forman et al. compared 112 babies born after letrozole therapy with 271 babies born after clomiphene citrate therapy and 94 newborns following spontaneous pregnancy. The rate of malformations was 0%, 2.6%, and 3.2%, respectively [32].

In general, teratogenic agents must be present during the period of embryogenesis (i.e. 18 to 54 day after fertilization) to cause congenital anomalies, on the other hand the exposure of embryo to teratogenic agents during the preimplantation period (i.e. 8 to 10 days after fertilization) does not cause congenital anomalies [33,34]. Because letrozole is completely cleared in five half lives after the last tablet is administered (i.e. about ten days), Casper suggested that letrozole administration in early follicular phase is not associated with any teratogenic effects because it is completely cleared before implantation[35]. In the present study, letrozole was stopped at least four days before IUI and therefore it was completely cleared at least two days before implantation.

## Conclusion

The data presented in our study indicated that the extended letrozole regimen had a superior efficacy as compared with clomiphene citrate in patients of unexplained infertility undergoing superovulation combined with IUI.

## List of abbreviations

BMI: Body mass index; FDA: Food and Drug administration; IUI: Intrauterine insemination; PCOS: Polycystic ovary syndrome; FSH: Follicle stimulating hormone; LH: Luteinizing hormone; E_2_: Estradiol; HCG: Human chorionic gonadotropin; OHSS: Ovarian hyperstimulation syndrome; HOXA10: Homeobox A10.

## Competing interests

The authors declare that they have no competing interests.

## Authors' contributions

Both authors have made substantial contributions to conception and design, or acquisition of data, or analysis and interpretation of data, and have been involved in drafting the manuscript or revising it critically for important intellectual content. Both authors have read and approved the final manuscript.

## Authors' information

Usama M. Fouda, MD, PhD, Lecturer of Obstetrics and Gynecology, Faculty of Medicine, Cairo university, and scientific director of assisted conception unit, Ahmed Elgazzar Hospital. Ahmed M. Sayed, MD, PhD, Assistant professor of Obstetrics and Gynecology, Faculty of medicine, Cairo university, and clinical director of assisted conception unit, Ahmed Elgazzar Hospital.
